# Scaling Our World View: How Monoamines Can Put Context Into Brain Circuitry

**DOI:** 10.3389/fncel.2018.00506

**Published:** 2018-12-20

**Authors:** Philipp Stratmann, Alin Albu-Schäffer, Henrik Jörntell

**Affiliations:** ^1^Sensor Based Robotic Systems and Intelligent Assistance Systems, Department of Informatics, Technical University of Munich, Garching, Germany; ^2^German Aerospace Center (DLR), Institute of Robotics and Mechatronics, Weßling, Germany; ^3^Neural Basis of Sensorimotor Control, Department of Experimental Medical Science, Lund University, Lund, Sweden

**Keywords:** monoamine neurotransmitter disorders, motor control, motor learning, neuromodulation, principal component analysis, raphe nuclei, serotonin, spinal cord

## Abstract

Monoamines are presumed to be diffuse metabotropic neuromodulators of the topographically and temporally precise ionotropic circuitry which dominates CNS functions. Their malfunction is strongly implicated in motor and cognitive disorders, but their function in behavioral and cognitive processing is scarcely understood. In this paper, the principles of such a monoaminergic function are conceptualized for locomotor control. We find that the serotonergic system in the ventral spinal cord scales ionotropic signals and shows topographic order that agrees with differential gain modulation of ionotropic subcircuits. Whereas the subcircuits can collectively signal predictive models of the world based on life-long learning, their differential scaling continuously adjusts these models to changing mechanical contexts based on sensory input on a fast time scale of a few 100 ms. The control theory of biomimetic robots demonstrates that this precision scaling is an effective and resource-efficient solution to adapt the activation of individual muscle groups during locomotion to changing conditions such as ground compliance and carried load. Although it is not unconceivable that spinal ionotropic circuitry could achieve scaling by itself, neurophysiological findings emphasize that this is a unique functionality of metabotropic effects since recent recordings in sensorimotor circuitry conflict with mechanisms proposed for ionotropic scaling in other CNS areas. We substantiate that precision scaling of ionotropic subcircuits is a main functional principle for many monoaminergic projections throughout the CNS, implying that the monoaminergic circuitry forms a network within the network composed of the ionotropic circuitry. Thereby, we provide an early-level interpretation of the mechanisms of psychopharmacological drugs that interfere with the monoaminergic systems.

## 1. Introduction

Metabotropic neuromodulators are ubiquitous in the CNS. Together with acetylcholine (Picciotto et al., 2012), the four monoamines serotonin (5-HT), dopamine, noradrenaline, and histamine dominate neuromodulatory effects in the CNS (Cools et al., 2011; O'Donnell et al., 2012; Yu et al., 2015). These molecules are strongly implicated in mood and affective state, while their malfunction is tightly linked to cognitive disorders (Kurian et al., 2011; Howell and Cunningham, 2015; Ng et al., 2015; Mather and Harley, 2016). A common view is that brain function emanates from signal processing of the ionotropic functional and anatomical connectome of the brain, which occurs with high topographic and temporal precision. In contrast to ionotropic neurotransmission, neuromodulation produces no direct excitatory or inhibitory effects mediated by the activation of the fast-acting ionotropic glutamate or GABA receptors. Instead, neuromodulation acts on G protein-coupled receptors and thereby changes the surface expression or efficacy of potassium, calcium, or sodium channels. This scales the general excitability, or gain, of the neuron (Haas et al., 2008; Rosenbaum et al., 2009; Beaulieu and Gainetdinov, 2011; Bargmann, 2012; Picciotto et al., 2012; Perrier et al., 2013; Husch et al., 2015; Perrier and Cotel, 2015). So far, monoamines are presumed to provide a diffuse general modulation of large connectome circuits (Fuxe et al., 2010). But knowledge of the specific functional contributions of monoaminergic neuromodulators to neuronal processing and the resulting integrative behavior is scarce.

In this paper, a novel functional principle is deduced for monoamines as temporal- and subcircuit-precise gain modulators. Whereas the ionotropic subcircuits can collectively signal predictive models of the world based on life-long learning, monoamines are shown to scale the influence of functionally distinct neuronal subcircuits individually. Hereby, their effects show just the right time constant to adjust the models to quickly changing contexts. By this precision scaling, monoamines provide an operation which may overcome functional limitations of ionotropic networks that apply under physiological conditions. The principle emerges from an analysis of monoaminergic effects in the specific context of locomotion, which integrates control theory of biomimetic robots, motor control neuroscience, and neurobiological findings on monoamine systems. Accordingly, serotonin must be assumed to scale motorpools of an individual joint when it shows particularly large movement, because this implies that the respective joint can be moved with smaller metabolic requirements. This precision scaling dramatically simplifies motor control adaptation in the face of gradually changing mechanical conditions which, for example, take place as one steps from a solid to a soft ground or lifts a load. But the principle of precision scaling is also tentatively applicable to general computational interactions between neuronal populations throughout the CNS and may thus support various high- and low-level functions.

Here we focus on the spinal motor circuitry to deduce if the CNS applies precision scaling. This focus has two reasons: First, it is comparatively easy to interpret how information is encoded and reconstruct how information is processed, because the spinal circuitry is the final motor output stage and the entry stage of low-level sensory feedback signals (Franklin et al., 2016). In contrast, higher-level systems operate by using more abstracted information that can be hard to interpret. Second, the control of body movement is widely assumed to be a major, if not the most important, factor for the evolution of the CNS (Wise and Shadmehr, 2002; Babič et al., 2016). This implies that cognitive levels evolved while being constrained by the spinal motor output and sensory input circuitry. Motor control can therefore be regarded as a basis to understand such higher integrative circuits.

In order to understand the spinal motor control, modern robotics control theory, which has been developed for robots with increasing functional similarity to biological locomotor systems, offers multiple advantages: Robotic control theory can provide comprehensive and well-tested analytical tools. If the major constraints of the CNS are taken into account, it further offers highly specific interpretational frames for understanding observations of sensorimotor control in the CNS. It goes without saying that early-stage testing of concepts for biological motor control is easier in robots than in biological systems.

In the present paper, we suggest that the mode of operation of various monoaminergic systems in the CNS is *precision scaling*, i.e., a topographically and temporally specific gain control of local neuronal operation. In the chain of argumentation that leads up to this prediction, we start out by comparing the functional operations within monoamine-driven metabotropic systems with those observed in ionotropic circuitry. Accordingly, the spinal ionotropic circuitry integrates descending motor commands and sensory signals and linearly processes them into muscle signals. By this function, it dominates the spinal generation of motor patterns, which has further contributions from gap junctions and diffuse metabotropic effects. Mathematically, the ionotropic circuitry transforms between the different representations, or the different “views,” of the world as they are encoded by the individual processing stages (section 2). Nonlinear signal processing is required to adjust these transformations to changing contexts. Based on neurophysiological findings, these non-linear adjustments are ideally solved by neuromodulatory scaling of the ionotropic signals due to the properties of the serotonergic system (section 3). The scaling effect renders the metabotropic system functionally unique, given that ionotropic effects proposed for non-linear signal processing in other CNS areas are unlikely to apply to spinal circuitry conditions *in vivo* according to recent experimental studies (section 4). Subsequently, the spatial and temporal precision of the spinal serotonergic system is evaluated to see if it may perform tasks that cannot be obtained by ionotropic circuitry under the influence of exclusively diffuse neuromodulation. Insights from robotic control and motor neuroscience are combined to identify such a task and deduce how focused serotonin must act, both anatomically and temporally, in order to solve it (sections 5, 6). This functionally required precision is demonstrated to coincide with the neuroscientifically observed topographic and temporal precision of serotonergic effects in the spinal cord (section 7). Monoamines must therefore be considered, at least partly, subcircuit- and temporally-specific gain modulators of ionotropic circuitry, motivating the term *precision scaling*. As will be shown toward the end of this paper, precision scaling can potentially apply to multiple levels of CNS function and may explain the effects of psychopharmacological drugs that act on monoamine systems in the brain (section 8).

## 2. The Spinal Cord as Transformer of World Views

The function of the CNS emanates from the neurophysiological processes in the individual neurons and the precise network of connections between them. In order to understand how monoaminergic neuromodulatory influences neuronal circuitry, it is important to first understand how functions arise in networks of ionotropic circuitry.

### 2.1. Linear Signal Processing Transforms How Spinal Neurons Encode the World

An individual neuron primarily works by integrating information in the form of the electrical signals it receives from other neurons by synaptic transmission. In response to the summated effect of those inputs, it issues electrical signals that reach other neurons. Hence, it can be said to process information. In the spinal cord, recent electrophysiological findings suggest that the neurons are hereby limited to linear processing of information encoded by ionotropic signals: Spinal interneurons which are subject to increasing single or multiple synaptic inputs respond linearly under physiological conditions *in vivo* (Prut and Perlmutter, 2003; Shalit et al., 2012; Spanne et al., 2014; Zelenin et al., 2015). In particular, they are active well before overt movement starts and do not saturate (Prut and Perlmutter, 2003), implying that they are in their linear regime during a movement. Investigations assign the same linear response to spinal motorneurons in anesthetized animals and *in vitro* (Powers and Binder, 1995, 2000; Hultborn et al., 2003; Cushing et al., 2005; Hyngstrom et al., 2008).

**Figure 1:**
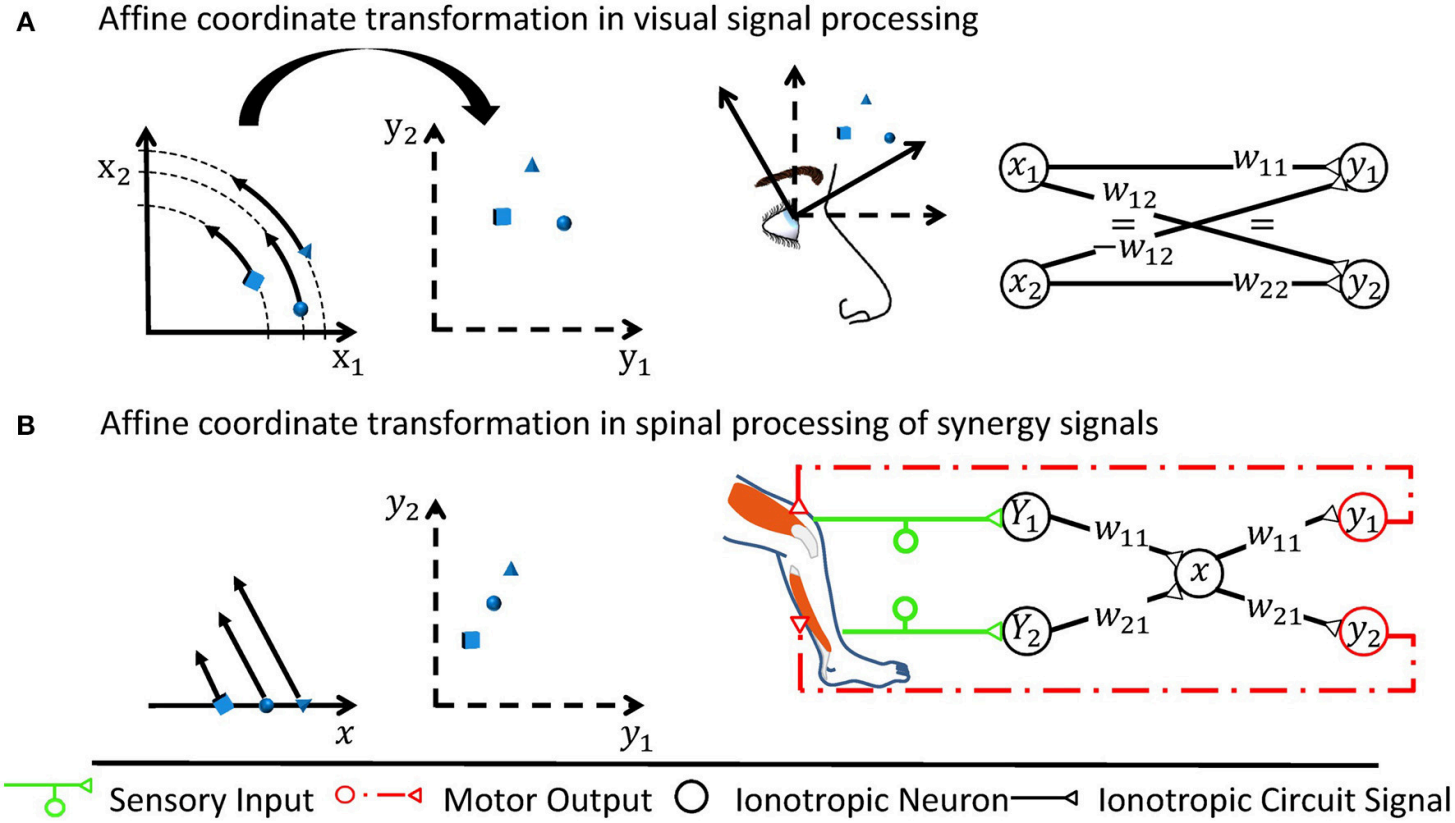
Two examples of affine transformations and their simplest neuronal circuitry connectivity solution. Each transformation is made from a coordinate space represented by neuronal states (*x*_1_, *x*_2_) or *x* to a space represented by (*y*_1_, *y*_2_), respectively. For rate-coded models, these neuronal states would be equivalent to firing rates of neurons or neuron pools. Sketches of neuronal networks show the simplest possible way by which the CNS might implement each transformation. As illustrated for each panel, the networks in effect re-calculate individual signals encoded by the neuronal states into new individual neuronal states, where the re-calculation is the specific transformation made and is illustrated by arrows in the leftmost graph. **(A)** A rotation is assumed to occur when neurons transform retinotopic neuronal signals occurring at early visual processing stages to a head-centered representation at later stages (Heed et al., 2015). **(B)** Shown here is the transformation underlying a motor synergy, i.e., a spinal circuit which emits common ionotropic signals to actuate muscles that the neuronal synergy models as agonists (Santello et al., 2016). The low-dimensional synergy signal *x* is thereby transformed into the high-dimensional musculotopic space represented by coordinates (*y*_1_, *y*_2_). Signals (*Y*_1_, *Y*_2_), such as sensory signals, can be transformed into this low-dimensional synergistic subspace by orthogonal projections.

As the CNS forwards information from one group of neurons to another, it filters out irrelevant aspects, combines data of different origin, and adjusts the way the information is encoded. All CNS functions can be traced back to such basal neuronal circuitry mechanisms. These basal mechanisms can be cascaded and integrated to create interesting local functions. The local functions can to a large extent be shaped by learning and can be regarded as partial, often predictive, models of the world, e.g., describing how photoreceptors are distributed across the retina or what motor signals must be elicited to perform a particular task (Brown and Brüne, 2012; Bhanpuri et al., 2013). What model of the world a neuronal circuit contains is determined by an associated coordinate space. For motor control, illustrative examples of coordinate spaces are found in visual reaching tasks: The target position is initially encoded as a pixelated image mapped in a retinotopic reference frame (Heed et al., 2015). To reach the target, the incoming visual signal requires neuronal processing and merging with additional information represented in non-retinotopic reference frames (cf. Figure 1). Processing generates an appropriate movement intention and an according signal in a musculotopic coordinate space at the level of the spinal motorneurons (Graziano, 2006; Yanai et al., 2008). Describing how a neuronal circuit encodes its associated coordinate space requires knowledge of the set of qualitatively relevant categories of encoded information. The categories may in principle be directly linked to physical quantities (Franklin et al., 2016), such as the activation of different muscles. But at integrative stages, they may also be linked to more high-level quantities like the social status and familiarity that an animal takes into account before approaching a potential partner. They may even be linked to highly abstract quantities with no direct counterpart in the physical world. Given appropriate coordinates, it is convenient to describe the information encoded in a group of neurons as a vector,

(1) $$\begin{array}{r} \text{x}=\left(\begin{array}{c} x_{1}\\ x_{2}\\ \vdots \end{array}\right)\;. \end{array}$$

Each coordinate *x*_1_, *x*_2_, … would in the mentioned examples describe the motor signal driving a single muscle or a component of a higher-level quantity encoded by the CNS. It can be represented by the signal of individual, or groups of, neurons (Cunningham and Yu, 2014).

Between coordinate spaces, linear neurons as found in the spinal cord perform affine transformations. Affine transformations, which are exemplified in Figure 1, are heavily used by engineers since they often approximate general transformations involving arbitrary mathematical functions very well for a limited range of input values around an operating point (Cohen and Tan, 2012). In a network consisting of linear neurons, each neuron *j* receives sensory inputs from presynaptic neurons *i*, which is represented by a firing rate *x*_*i*_ for rate-coding neurons. The inputs are weighted by synaptic weights *w*_ij_ and subject to a neuron-specific firing threshold θ_*j*_. In summary, the output firing rate *y*_*j*_ in neuron *j* can be described by the linear function

(2) $$\begin{array}{r} y_{j}\left(x_{1},x_{2},\ldots \right)=\underset{i}{\sum }w_{\text{ij}}\cdot x_{i}-\theta_{j}. \end{array}$$

Equation (2) can be represented in vector notation as

(3) $$\begin{array}{r} \text{y}\left(\text{x}\right)={\text{W}}_{\text{xy}}\cdot \text{x}-{\text{θ}}_{\text{y}}. \end{array}$$

It defines the affine transformation between two coordinate spaces in which the signal can be represented by coordinates **x** and **y**, respectively.

**Figure 2:**
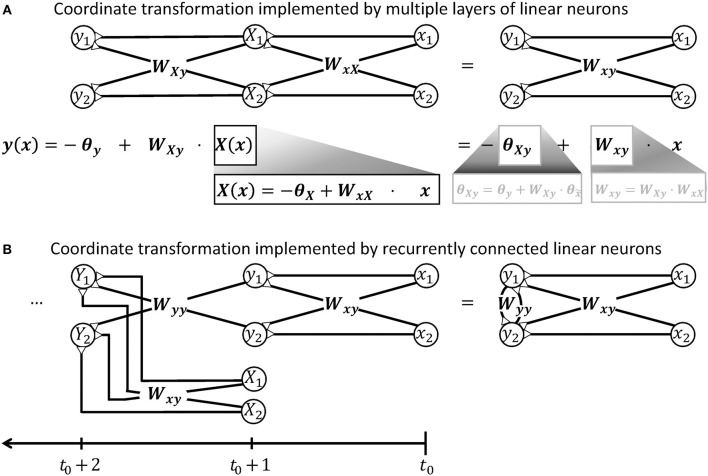
Illustration demonstrating that a network of linear neurons is restricted to implement an affine coordinate transformation of the form **y**(**x**) = **W**_xy_**x**−***θ***_**y**_. **(A)** This relationship is independent of the number of incorporated neuronal feedforward layers. Adding additional layers of neurons changes the input-independent transformation weight **W** and the shift ***θ*** of the basis, but the mathematical form remains. **(B)** Neuronal networks with recurrent connections are subject to the same limitation. To confirm this, it is advantageous to unfold the calculations performed by the recurrent network shown on the right hand side and deduce a hypothetical feedforward network that computes the same output. When the recurrent network receives an input signal **x** released at *t*_0_, it will produce an output signal **y** like a simple feedforward network at *t*_0_+1, i.e., after a short unitary transduction delay. At the next computational step *t*_0_+2, the output signal **Y** is determined by the input signal **X** from time step *t*_0_+1 and the previous output signal **y**. The previous output signal is thereby feed back by recurrent synapses with weights **W**_yy_. To model this recurrent calculation, one may extend the hypothetical feedforward network by a further layer of linear neurons as shown on the left hand side. These neurons receive the previous output **y** via synaptic weights **W**_yy_. They also receive the further input signal **X** from time step *t*_0_+1 from an additional pool of input neurons which synapse via synaptic weights **W**_xy_. Further calculation steps *t*_0_+3, *t*_0_+4, …, *t* of the recurrent network can be modeled in the feedforward network by iteratively adding layers with the same properties. Thus, the output **y** of the recurrent network after *t* time steps is mathematically equivalent to the output produced by a hypothetical feedforward network with *t*−1 intermediate and one output layers. According to the argument in the beginning of this caption, this multi-layered feedforward network implements an affine coordinate transformation. During each individual time step, also the recurrent network can thus only perform an affine coordinate transformation on its input.

A spinal ionotropic network consisting of linear neurons is limited to implement an affine transformation which can be described by Equation (3). Neither additional feedforward layers, nor recurrent synaptic connections can change this functional property. If the neuronal network is extended by intercalating further layers of linear neurons between the input and output layer, only the effective transformation weights **W**_xy_ and threshold ***θ***_y_ of the transformation will change. As shown in Figure 2A, the output firing rate will remain a linear function of the network input. If the network is extended by recurrent synaptic connections, it can memorize input and process time-series of data. Thereby, its output may vary non-linearly with time and, for example, converge to a steady state or oscillate (Dayan and Abbott, 2001). But at each time step, the network output **y** remains a linear function of its previous input **x** at previous time steps, as illustrated in Figure 2B.

### 2.2. Non-linear Signal Processing Is Required When the World Changes

Artificial neural networks have, with the enormous scientific and economical success of deep learning in particular and of artificial intelligence in general, strongly facilitated the view that also biological neuronal networks can approximate general transformations which adjust the output of the network to arbitrarily changing conditions (Chen et al., 2015). In mathematical terms, they are said to perform universal classification and function approximation. This view relies on the model of neurons as non-linear integrators of incoming signals (Cybenko, 1989; Hornik, 1991). While this contrasts the observed linear interaction of ionotropic signals for the specific example of the spinal circuitry, it must be assumed that also the spinal cord needs mechanisms which non-linearly combine external signals with the ionotropic inputs that the neuronal network processes. This becomes particularly obvious under quickly changing mechanical conditions of the environment and the locomotor system. Hereby, the changing context often requires that the CNS reacts differently to the same inputs. The multiplicative transformation weights of Equation (2) must therefore be context-dependent and change with a signal *s* which encodes the external cue,

(4) $$\begin{array}{r} y_{j}\left(x_{1},x_{2},\ldots ,s\right)=\underset{i}{\sum }w_{\text{ij}}\left(s\right)\cdot x_{i}-\theta_{j}\;. \end{array}$$

This implies a non-linear integration of the signals *s* and *x*_*i*_. In contrast, adding the signal *s* as an additional linear input, e.g., by a reflex loop that signals *s* and also converges onto the neurons *j*, would only additively increase the output of the network. In effect, it would only change its firing threshold *θ*_*j*_.

**Figure 3:**
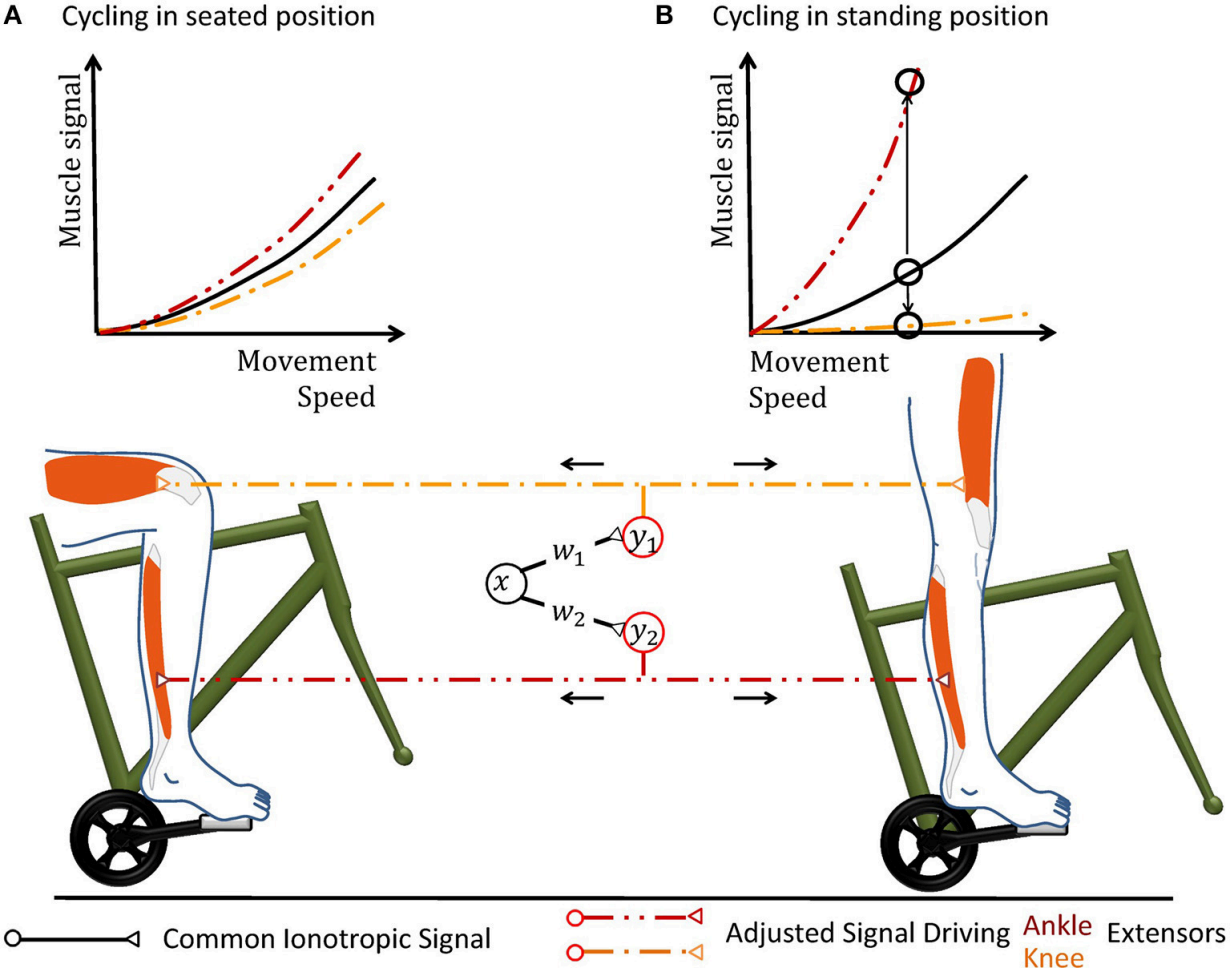
Cycling can illustrate why a hypothetical coordinate transformation requires topographically precise non-linear scaling under varying conditions. The pedal forms a mechanical environment that constrains the movement and the muscular forces required to excite it in a particularly strict way. In the hypothetical neuronal circuit, a group of neurons carries a motor signal *x* that triggers several groups of muscles. As the motor signal is forwarded to spinal interneurons and motorneurons, it is transformed according to the transformation weights *w*_j_. The resulting musculotopic signals *y*_j_ simultaneously actuate muscles involved in pushing the pedal, e.g., extensor muscles of the knee and ankle. Increasing the movement speed can be performed by an overall increase of the common motor signal. **(A)** When the person sits on the saddle, both knee and ankle extensors need to be actuated simultaneously. The weights of the synapses transferring the hypothetical motor signal *x* to the respective motorneuron pools are hereby chosen accordingly to achieve the required respective muscular signals. **(B)** When the mechanical properties of the environment changes during an ongoing movement, e.g., because the subject stands up to accelerate, the same muscle groups need to alter their output during the same phase of locomotion. In the standing position, knee actuation cannot exert force along the pedal trajectory. Thus, knee extensors should receive almost no muscle signal, while ankle extensors need to be activated more in order to keep up a given movement speed. Ankle and knee extensors thus need a scaling of different polarity, as illustrated by the upwards and downwards arrow in the graph. Under changing mechanical conditions, all motorneuron pools can thus still be actuated by the same abstract ionotropic motor signal from the hypothetical neuronal synergy, which just needs to be scaled differentially according to sensory information.

By adjusting individual transformation weights independently from each other, the motor circuitry can gain a unique functionality. Figure 3 illustrates this functionality based on the transformation of context-dependent motor signals from M1 onto the musculotopic motor output within spinal circuits. Hereby, pools of M1 neurons typically elicit a common motor command which is transformed into musculotopic signals as it is transmitted to spinal motorneurons either directly or through spinal interneurons (Yanai et al., 2008). The transformed motor command activates the spinal motorneuron pools of several muscles to produce a meaningful pattern of muscle contraction (Graziano, 2006; Overduin et al., 2012; Gallego et al., 2017). In this circuit, the transformation weights along the path between M1 and the motorneuron pools will need to be scaled in a pool-specific manner if a new mechanical condition necessitates that the involved muscles change their force output relative to each other. Similar examples for neuronal operations that require changing transformation weights can be found in integrative circuits such as the ventral intraparietal area. This area encodes an abstract representation of vestibular self-motion signals that is independent of head and eye position (Chen et al., 2013). To decouple vestibular signals from head and eye movement, the transformation of vestibular signals onto intraparietal neurons must be adjusted according to time-varying signals encoding the motion (Salinas and Sejnowski, 2001). While the ionotropic signal processing is shaped by synaptic plasticity, it is important to notice that its non-linear adjustment during ongoing motor control inherently differs from synaptic plasticity rules in two ways. First, synaptic plasticity acts on a time scale of minutes to hours in motor circuitry (Nishimura et al., 2013), which is too slow for adjustments to changing mechanical contexts. Second, synaptic plasticity is typically local (Gerstner, 2016), whereas the external signal *s* modulates the transformation weights between multiple pre- and postsynaptic neurons that encode input signals *x*_*i*_ but not the signal *s* itself. The spinal cord needs to implement such a mechanism which non-linearly integrates signals in order to adjust transformations between neuronal information at different stages of processing, or abstraction, according to changing context.

## 3. Monoamines Scale Signals in Spinal Motor Circuits

How would such a non-linear signal integration occur? Functional and anatomical evidence suggests that, in contrast to ionotropic receptors, metabotropic neuromodulation enables non-linear signal integration within spinal motor circuits. A serotonergic signal *s* is thereby a promising candidate for adjusting the spinal signal processing in line with Equation (4), as it can encode the changing context.

Serotonin (5-HT) released within the ventral spinal cord increases the gain or response of both spinal motorneurons (Hochman et al., 2001; Heckman et al., 2008) and ventral spinal interneurons (Abbinanti and Harris-Warrick, 2012; Abbinanti et al., 2012; Husch et al., 2015; Perrier and Cotel, 2015) to ionotropic input, without affecting their baseline excitation. This effect is functionally equivalent to an increase of the transformation weights onto motorneurons. It results from a stimulation of 5-HT_2_ receptors, which triggers a range of biochemical mechanisms as extensively reviewed previously (Abbinanti and Harris-Warrick, 2012; Perrier et al., 2013). Stimulating 5-HT_2_ receptors by descending 5-HT is crucial in particular for the generation of rhythmic movement in mammals, such as whisking in rats (Hattox et al., 2003) and weight-supported locomotion (Slawińska et al., 2014). By activating 5-HT_1A_ receptors, the CNS can in turn divisively scale down the transformation weights of ionotropic circuitry converging onto motorneurons. The underlying decrease of motorneuronal gain has been suggested to occur during muscle fatigue, when 5-HT spills over its synaptic release site after prolonged release and diffuses to the axon initial segment (Cotel et al., 2013). Before fatigue occurs, the CNS can scale up the firing rate of spinal neurons monotonously and multiplicatively by a factor of up to 10 by increasing the concentrations of 5-HT (Heckman et al., 2008).

**Figure 4:**
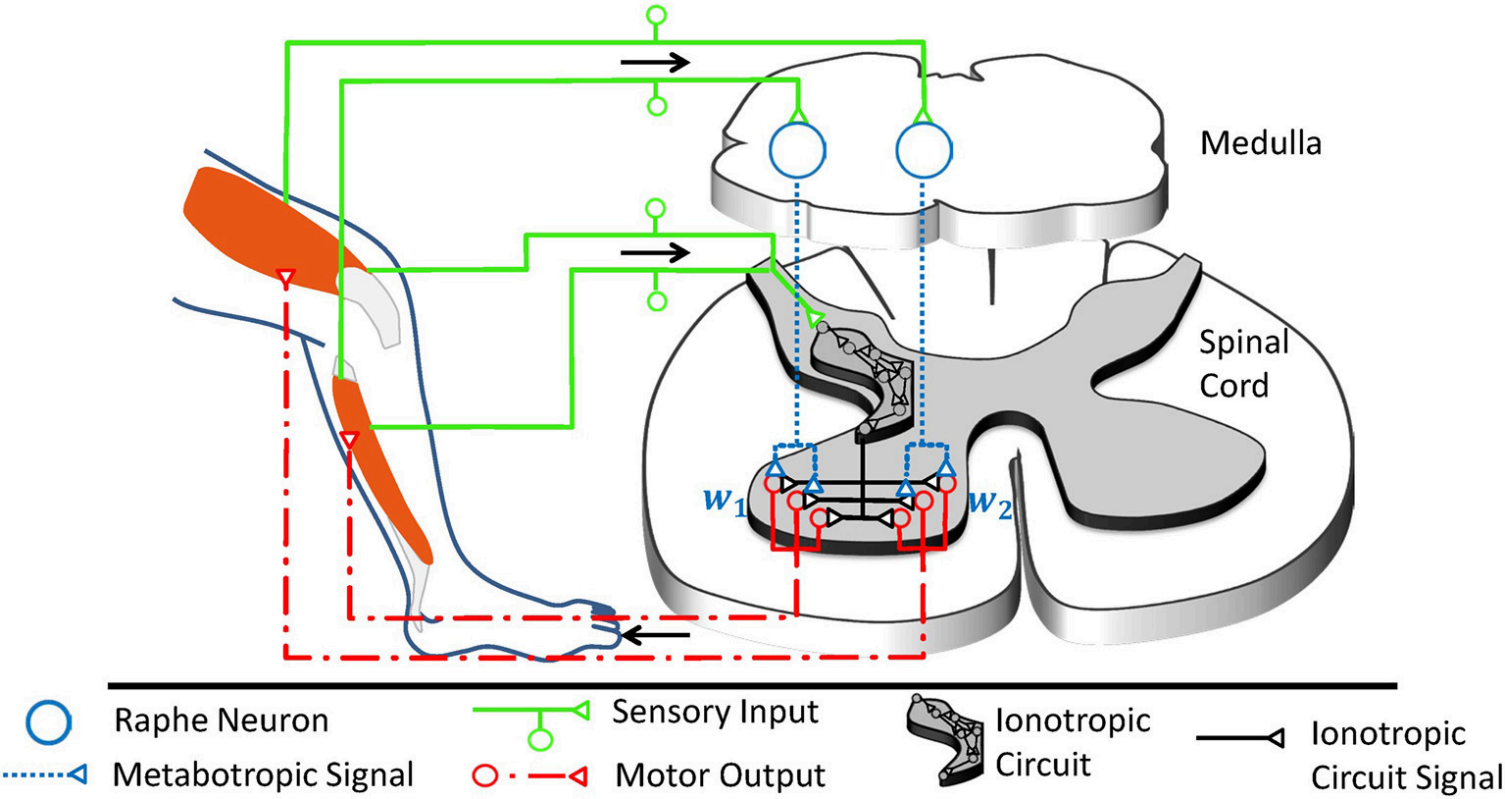
The raphe nuclei obscurus and pallidus form a motor feedback loop. They receive proprioceptive signals and accordingly release serotonin into the ventral spinal cord. The resulting higher serotonin concentration metabotropically increases the excitability of spinal motorneurons as well as associated interneurons at the motor output stage. Collectively, these serotonergic raphe projections change the relative multiplicative weights *w*_j_, which describe how ionotropic signals are transformed into musculotopic motor output. The serotonergic feedback loop acts in parallel to the ionotropic processing of sensory signals. Ionotropic circuitry could operate independently of the metabotropic weight adjustments at the motor output stage and could implement, for example, a low-dimensional control circuit as illustrated here and in Figure 1B. Those projections of the raphe nuclei obscurus and pallidus which target interneurons within the low-dimensional circuit will scale the overall spinal ionotropic motor signal without affecting the relative strength *w*_j_ of the signals actuating different muscles. In this figure, the ionotropic circuit and each motorneuron pool are represented by several neurons, which appear to be functionally redundant. However, neurons within a subcircuit may have dissimilar connections that assign them to different subcircuits during other tasks. Figure modified, with permission, from Stratmann et al. (2016).

In the ventral spinal cord, neuromodulatory effects are dominated not only by 5-HT, but also by noradrenaline (Heckman et al., 2008) and neuropeptides (Thörn Pérez et al., 2015). Neuropeptides are co-released with monoamines and partly trigger similar biomechanic mechanisms (Thörn Pérez et al., 2015), but their predominant trophic effects are very slow (Svensson et al., 2001). In contrast to noradrenaline, 5-HT particularly stands out as candidate for multiplicative operations governed by a mechanical context, as serotonergic neurons receive proprioceptive information on a given movement and implement a distinct motor feedback loop as illustrated in Figure 4. About 90% of the 5-HT present within the spinal cord originates from the raphe nuclei (ElBasiouny et al., 2010). In the ventral spinal cord, 5-HT originates primarily from the nucleus raphe obscurus (NRO) and pallidus (NRP), which in turn project almost exclusively to the ventral spinal motor circuitry (Martin et al., 1978; Loewy, 1981; Nieuwenhuys et al., 2008; Watson et al., 2012). These medullary nuclei receive proprioceptive inputs, potentially including inputs from cutaneous mechanoreceptors, and increase the firing rate of their serotonergic neurons accordingly (Springfield and Moolenaar, 1983; Veasey et al., 1995; Fornal et al., 1996). In agreement with the concept of a motor feedback loop, ionotropic motor output is functionally facilitated by 5-HT as an after-effect following strong muscle contraction (Crone et al., 1988; Wei et al., 2014).

There is one pivotal caveat to the presented concept of serotonin as the modulator of individual transformation weights in the ionotropic processing of information: Monoamines are typically considered to be slow and diffuse modulators of a spatially and temporally precise ionotropic circuitry. In fact, the ventral spinal serotonergic system will have a topographically diffuse effect on motor output for the reason that it partly projects to spinal interneurons, which often target several groups of muscles simultaneously (Santello and Lang, 2015; Pérez-Nombela et al., 2017; Takei et al., 2017). The diffuse component will scale the overall Spinal ionotropic motor signal without affecting the relative strength of signals actuating different muscles. But as will be detailed below, recent work suggests that the ventral spinal projections of the NRO and NRP have also a topographically specific component which performs precision scaling (Stratmann et al., 2016). In the following sections, the chain of argumentation will demonstrate that previous findings on the described serotonergic motor feedback loop are consistent with a role as a functionally specific multiplicative operator. By this precision scaling, the raphe nuclei accordingly overcome the limitations of ionotropic circuitry. The arguments run along three lines: First, metabotropic systems are shown to offer a unique functionality in the spinal cord, since ionotropic mechanisms cannot implement non-linear interaction of signals in this CNS region. Second, a fundamental motor control task is considered to define what spatial and temporal precision the serotonergic system needs in order to offer a meaningful functionality that cannot be obtained by diffuse neuromodulation. For this purpose, the particular affine transformation involved in synergy control is chosen as the system of study, as it is both likely implemented by spinal ionotropic circuits and solves motor tasks that would benefit from a subcircuit-specific gain-scaling mechanism. Third, the functionally required spatial and temporal precision will be compared with the experimentally observed precision of the serotonergic feedback loop.

## 4. Limitations of Ionotropic Signal Interaction *in vivo*

The adjustment of coordinate transformations to external signals could theoretically be performed by a neuronal network using solely ionotropic synaptic currents. Based on neurophysiological findings, several mechanisms have previously been proposed for non-linear, particularly multiplicative, interactions of ionotropic signals. They are typically linked to specific respective CNS regions, have recently been reviewed in detail (Silver, 2010; Carandini and Heeger, 2012) and are summarized in Table 1. As mentioned before, the spinal interaction of ionotropic signals is known to be highly linear. This can be attributed to the properties of spinal neurons and signals, which make mechanisms suggested for other CNS regions physically implausible and typically even impossible.

**Table 1:** Overview of various mechanisms proposed for multiplicative interaction of neuronal signals.

**Coding regime mechanism**	**Explanation**	**References**
Time-sparse encoding		
Synaptic noise from balanced excitatory and inhibitory input	Noise triggers membrane voltage to cross threshold by chance and thus smooths input-output function around spiking threshold.	Pyramidal neurons from somatosensory cortex: Chance et al. (2002); Higgs et al. (2006). Motoneurons from spinal cord: Berg et al. (2007).
Shunting inhibition	Inhibitory input in proximity to the soma increases the membrane conductance, which divisively scales the postsynaptic potentials	Theoretical explanation: Isaacson and Scanziani (2011). Neurons from lateral geniculate nucleus: Sherman and Koch (1986). Granule neurons from cerebellum: Mitchell and Silver (2003).
Rate-based encoding		
Short-term synaptic depression	Divisively scales input when a further signal is added.	Neurons from V1: Carandini et al. (2002); Ozeki et al. (2009); Carandini and Heeger (2012). Granule cells from cerebellum: Rothman et al. (2009).
Logarithmic signals	Multiplication of signals *x*_*i*_ turns into a summation upon their logarithmic transformation: log(*x*_1_·*x*_2_) = log(*x*_1_)+log(*x*_2_) .	Locust lobula giant motion detector: Jones and Gabbiani (2012).
Active dendrites	Voltage dependent channels can induce sub- or supralinear signal interaction.	Theoretical explanation: Segev et al. (1994); Rhodes (2006). Pyramidal neurons from sensorimotor cortex: Oviedo and Reyes (2002); Williams and Stuart (2002). Pyramidal neurons from hippocampus: Losonczy and Magee (2006); Remy et al. (2009). Pyramidal neurons from neocortex: Major et al. (2013).
Monoaminergic neuromodulation	Activation of G protein-coupled receptors changes neuronal excitability.	Review on general monoaminergic functions: Bargmann (2012). Review on dopamine receptors: Beaulieu and Gainetdinov (2011). Review on cholinergic receptors: Picciotto et al. (2012). Serotonergic receptors in the spinal cord: Perrier et al. (2013); Husch et al. (2015); Perrier and Cotel (2015); this paper.

Mechanisms of multiplicative signal interactions can be split into two groups (Silver, 2010): Some mechanisms work in neurons which show time-sparse encoding, i.e., which encode data in the correlations of spikes. Other mechanisms apply to neurons which show a rate-based encoding of information, implying that the neurons process information by exploiting a large range of firing rates.

For neuronal networks working in temporally sparse coding regimes with low firing rates, two main mechanisms for non-linear interaction have been proposed. The first is based on changing levels of synaptic noise emerging from balanced excitatory and inhibitory input (Berg et al., 2007), which can change the gain of the input-output function for neurons operating around their spiking threshold (Chance et al., 2002; Mitchell and Silver, 2003; Higgs et al., 2006). The second is based on shunting inhibition produced by inhibitory input in spatial proximity to the soma (Sherman and Koch, 1986; Isaacson and Scanziani, 2011). These mechanisms are unlikely to cause gain scaling in spinal circuitry, where the early sensory processing and motor output are dominated by rate-coded signals under normal behavior (Ahissar, 1998; Maier et al., 1998; Perlmutter et al., 1998; van Rossum et al., 2002; Stein et al., 2005; Shalit et al., 2012).

For neurons that work within a rate-coded regime, non-linear signal interaction can occur due to the short-term synaptic depression (STD) of synaptic efficacy. If a neuron transmits the sum of two excitatory signals, the second signal may push the firing rate into a regime where STD occurs and may therefore divisively scale the circuit response to the first signal (Carandini et al., 2002; Ozeki et al., 2009; Rothman et al., 2009; Carandini and Heeger, 2012). Using this mechanism is metabolically unfavorable compared to other possible non-linear mechanisms, as the neuronal network would transmit a particularly high number of metabolically expensive action and synaptic potentials (Magistretti and Allaman, 2015). In addition, recent recordings on rate-coding neurons which carry sensor and motor signals show that STD only takes place at the onset of a stimulation train (McElvain et al., 2015). During sustained firing, STD was found to saturate and remain constant for a wide range of firing rates. Thus, STD is unlikely to occur in spinal calculations during ongoing behavior. A second hypothesis originates from the mathematical fact that the multiplication of two signals turns into a pure addition when the logarithms of the signals are considered,

(5) $$\begin{array}{r} \log\left(x_{1}\cdot x_{2}\right)=\log\left(x_{1}\right)+\log\left(x_{2}\right)\;. \end{array}$$

For signals which are encoded logarithmically, such as specific quantities in the visual systems (Gabbiani et al., 2002; Jones and Gabbiani, 2012), multiplication thus becomes trivial (Jones and Gabbiani, 2012). However, many mechanical stimuli are known to be linearly encoded by sensory firing rates (Hensel, 1973; Davis, 1975; Rothwell, 1987; Muniak et al., 2007; Bensmaia, 2008). Furthermore, a neuronal network which implements this strategy would be restricted to implement exclusively either multiplicative or additive operations on its inputs. To implement both, it would need to implement an additional exponential function to extract the actual coordinates. The third possible non-linear mechanism uses active properties of dendrites. Voltage-dependent Na^+^ and Ca^2+^ channels as well as NMDA receptors can individually induce supralinear and sublinear interaction of ionotropic signals (Oviedo and Reyes, 2002; Williams and Stuart, 2002; Mehaffey et al., 2005; Losonczy and Magee, 2006; Rhodes, 2006; Remy et al., 2009; Major et al., 2013). In concert, the non-linear effects can counteract the sublinear integration of signals caused either by passive dendritic properties (Segev et al., 1994) or by other voltage-dependent channels (Mehaffey et al., 2005; Rhodes, 2006; Palmer, 2014). The resulting overall effect is strongly determined by the clustering properties of converging synaptic inputs. Individual non-linear effects of unclustered inputs typically balance out to a linear signal summation (Priebe and Ferster, 2010). And indeed, *in vivo* mappings of the full dendritic tree of neurons at early sensory stages demonstrated that synaptic input is not clustered according to functional similarity, a finding which is consistent across different sensory systems (Jia et al., 2010; Varga et al., 2011). In agreement, other *in vivo* recordings showed that the individual non-linear effects of active dendrites are highly balanced and in effect facilitate a linear relationship between input current and output firing (Cash and Yuste, 1998, 1999). The same balance was found for spinal motorneurons in simulations (Cushing et al., 2005) and experiments (Hyngstrom et al., 2008) when neuromodulatory metabotropic input was removed. *In vivo* experiments on non-linear input summation of input from both eyes further emphasized that the CNS uses active dendritic properties not as a non-linear operation, but as a linearizing agent in sensory systems. The non-linear summation of individual signals was found to ensure that the output to binocular stimulation equals the linear summation of input during monocular stimulation (Longordo et al., 2013).

In conclusion, the specific physiological conditions of the spinal cord explain and emphasize that spinal neurons are linear integrators of incoming ionotropic signals. Therefore, the spinal cord needs to take advantage of the metabotropic serotonergic system in order to implement a non-linear interaction of signals.

## 5. Lightening the Burden of Freedom

### 5.1. Synergies Simplify the Control of Redundant Locomotor Systems

In order to understand how serotonergic precision scaling can improve motor behavior, it is necessary to consider a typical coordinate transformations implemented by ionotropic spinal circuitry.

Synergies are an example of spinal transformations which neuroscientists have analyzed in detail. They are formed by interneurons that either receive many input signals or project to motoneurons of several muscles. As illustrated in Figure 1B, the CNS thereby transforms high-dimensional sensory information into the low-dimensional synergy space and transforms the motor output from the synergistic circuitry into the high-dimensional musculotopic space (Lacquaniti et al., 2012; Alessandro et al., 2013; Santello et al., 2016). The input sensory synergy filters out information which is unnecessary for a specific motor task. It therefore chooses a particular combination of sensory information from the infinite combinatorial possibilities of sensory signals. The output motor synergy predetermines coordinated activation of a group of muscles elicited by a single circuit. It allows the CNS to choose from an infinite number of possible movement patterns in a locomotor system with more degrees of freedom than is required for a specific task (Bernstein, 1967). The human hand is the most obvious example for such a redundant mechanical system (Santello et al., 2016). But also each lower human limb comprises more than 50 muscles which are to a major extent recruited together for locomotion (Lacquaniti et al., 2012). This redundancy provides a high versatility of possible movements.

Behaviorally, neuronal synergies become detectable as a spatiotemporal pattern of EMG signals emanating from different muscles. They can be extracted mathematically by linear source decomposition methods like principal component analysis (Naik et al., 2016). Human locomotion shows four to five basic patterns (Lacquaniti et al., 2012), which are reproducible when locomotion is perturbed (Chvatal and Ting, 2012). To change locomotion speed, their relative recruitment is shifted gradually (Hagio et al., 2015).

Neurophysiological analysis indicates that the spinal cord is an important basis for synergy control (Santello et al., 2013; Jörntell, 2016; Kiehn, 2016). The majority of spinal interneurons combine signals from different modalities into sensory synergies (Jankowska, 1992). In turn, groups of interneurons elicit a synergistic pattern of muscle activations (Clark et al., 2010; Levine et al., 2014; Danner et al., 2015; Santello and Lang, 2015; Pérez-Nombela et al., 2017; Takei et al., 2017). Synergy output is thereby transformed into the high-dimensional musculotopic space and may be further routed through a separate neuronal layer before it reaches the motoneurons (Zhong et al., 2012). The cerebellum links the individual synergies into more elaborate synergies or into sequential patterns (Bengtsson and Jörntell, 2014; Jörntell, 2017). Descending cortical motor commands may accordingly excite individual synergies to produce meaningful, complex behavior (Graziano, 2006; Overduin et al., 2012; Gallego et al., 2017). These commands may in fact be partly transferred by diffuse neuromodulation, which is known to activate movement patterns or increase the movement frequency (Jing et al., 2009; Harris-Warrick, 2011). In summary, the evidence implies that spinal interneurons often combine information in a low-dimensional synergy space, and the synergistic muscle output is transformed and forwarded to the redundant locomotor system.

Understanding synergies is essential for studies on integrative motor circuits. They can be regarded as a library of re-usable modular building blocks, which the brain combines in order to construct a large range of complex learned and new movements from basic old ones. In the low-dimensional synergy space, the brain can integrate descending pathways, reflexes, and central pattern generators, i.e., neuronal circuits which produce rhythmic movement without rhythmic input (Ijspeert, 2008; Guertin, 2013; Ijspeert et al., 2013; Dzeladini et al., 2014; Kiehn, 2016; Minassian et al., 2017).

### 5.2. Robotics Control Theory Explains Synergistic Motor Control

Functional insights on the use of synergies can be obtained from robotics control theory. In recent work, an artificial neural network, which formed a similar network structure as a sensorimotor synergy, was trained to encode meaningful motor primitives within the intermediate synergy layer (Chen et al., 2015). The underlying type of artificial neural networks is called *autoencoder* and is typically used in in the field of deep learning to reduce the dimensionality of data (Hinton and Salakhutdinov, 2006). But while autoencoders in general deploy non-linear quasi-ionotropic mechanisms in the simulated neurons, also the use of more biologically plausible linear synergy spaces have been functionally well-examined for the control of biomimetic robotic hands (Bicchi et al., 2011; Santello et al., 2016) and legged systems (Aoi et al., 2017; Lakatos et al., 2017). The tools that have been developed in this endeavor are mathematically advanced and well-tested. Neuroscientists can thus use them to functionally explain or even predict a specific synergistic behavior.

For low movement speeds, the robotic control strategy of null space projections explains how several tasks, which are individually solved by a respective synergy, can be executed simultaneously (Dietrich et al., 2015). The top-priority command is executed using the full capability of the locomotor system, such as a synergy responsible to keep balance. If the locomotor system is redundant for that specific task, a lower-priority task, e.g., defined by a secondary synergy, can be executed to the greatest possible extent as long as it does not interfere with the top-priority task. For this purpose, an affine transformation projects the secondary task into a space formed by the redundant degrees of freedom, and the resulting motor signal is added to the signal of the top-priority command. The transformation weights depend on the current positions of the actuators, and their adjustments requires a precise model of the locomotor system and its environment (Featherstone and Khatib, 1997). The need for precise models applies to most strategies devised to control low movement speed (Braun et al., 2011). To adjust complex movements at low movement speed to changing conditions, it is therefore likely that human neuronal control circuits also require precise models of their locomotor system. Accordingly, the underlying circuits require high topographic precision. Since the fine-control of complex slow movements strongly relies on the supraspinal circuitry (MacKay-Lyons, 2002; Shemmell et al., 2009), it is reasonable to assume that the required precise models are encoded in the more sophisticated supraspinal neuronal networks. The slow movement speed allows for a heavy recruitment of these networks despite their long feedback delays.

### 5.3. Synergies for the Control of Highly Dynamic Movement

Here, we want to define a minimal precision that serotonergic effects need to show in order to perform a task that cannot be explained by diffuse neuromodulation. This suggests considering control strategies that require little model precision. It is likely that the CNS recruits such strategies more during fast and strong movements like running. These are strongly shaped by the inertia and elasticity of the system, i.e., quantities which can only be modeled with high inaccuracies and change over time (Nakanishi et al., 2008; Peters et al., 2008; Dietrich et al., 2015). Biomechanical locomotor systems are substantially more complex than robotic systems, as their dynamics critically depend on a particularly large range of parameters such as non-linear muscle elasticities, hysteresis effects, and the changing muscular 3D structure (Siebert and Rode, 2014). This emphasizes that control strategies which require a minimal model precision can control highly dynamic movements in biomechanical locomotor systems much more robustly than model-dependent strategies. A second advantage of considering the control of highly dynamic movement is the associated high consumption of metabolic energy within muscles. The metabolic demands can be drastically lowered by a control strategy utilizing elastic elements within tendons and muscles, as these elastic elements store kinetic energy during a ground impact and release it for recoil (Holmes et al., 2006; Sawicki et al., 2009; Lai et al., 2014). During the evolutionary development of the CNS, strategies for the energy-efficient control of this movement type were thus most likely a critical selection factor. For these reasons, control strategies for highly dynamic movements are likely to be implemented by the CNS. They require a minimum of model knowledge and are thus promising to estimate the minimum precision that serotonergic effects need to show.

**Figure 5:**
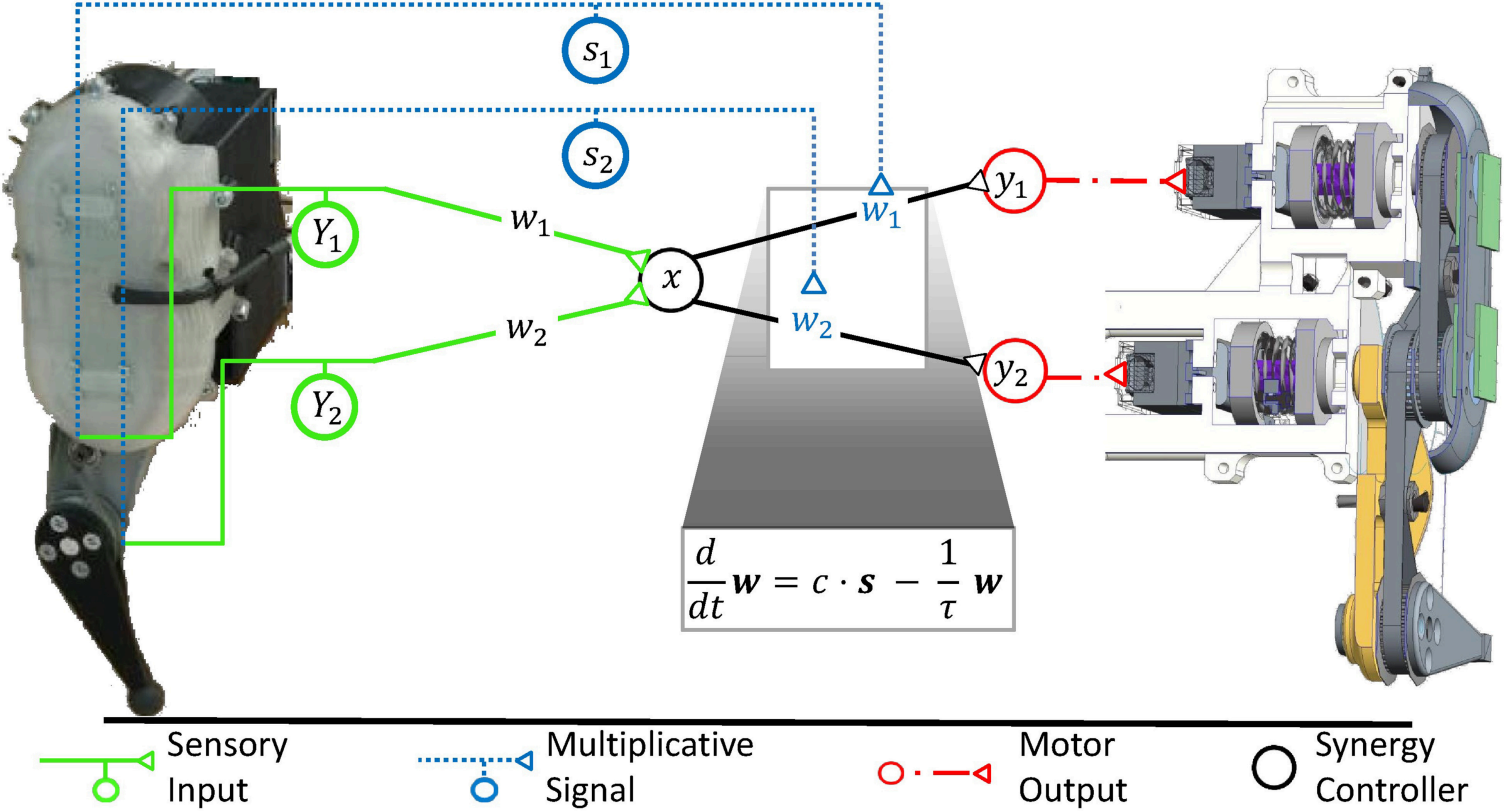
The robotic controller, which was previously developed to maintain a stable, fast, and strong movement, is mathematically equivalent to a synergy controller as illustrated in Figure 1B. Sensory input (*Y*_1_, *Y*_2_) signals the movement of individual degrees of freedom of the mechanical system. It is transformed into the low-dimensional synergy space and adjusts the phase and frequency of a synergistic motor signal *x*. Since the different mechanical degrees of freedom move with high synchrony during fast locomotion, the input weights can be chosen arbitrarily without loss in movement performance (Stratmann et al., 2016). The motor signal *x* is reversely transformed along the weights (*w*_1_, *w*_2_) to drive the actuators of the same robot. These weights are functionally critical, as the relative forces (*y*_1_, *y*_2_) of different actuators determine, among others, how well the robot can take advantage of its elastic elements to store energy upon ground impact and release it for recoil. In robotic systems, springs typically dominate the elastic properties of the system, as shown on the right hand side of this figure in a cross-sectional illustration of the exemplary robot. As detailed in section 6, the synergy controller can maintain an elastic movement with optimal energy efficiency under changing mechanical conditions. For this purpose, the controller receives sensory inputs *s*_1_ and *s*_2_, which signal the deflections of the degrees of freedom such as joint angles. To adjust to changing mechanical conditions, the controller needs to multiplicatively scale the output transformation weights *w*_1_ and *w*_2_ according to these inputs *s*_1_ and *s*_2_, respectively (Stratmann et al., 2017). A common multiplicative factor *c* determines the jumping height or distance. To keep the weights bounded, they need to decay exponentially with a time constant τ. The ventral spinal serotonergic system forms a motor feedback loop, as illustrated in Figure 4, which functionally resembles the loop of the multiplicative signals presented here.

For highly dynamic motion, robot control theory showed that a simple synergy controller can generate movement which is stable (Lakatos et al., 2013; Lakatos and Albu-Schäffer, 2014a,b) and makes optimal use of elastic elements in the locomotor system to minimize the consumption of metabolic energy (Stratmann et al., 2017). According to this control strategy, sensory information is linearly transformed into the one-dimensional synergy coordinate space, where it periodically drives a synergy controller (cf. Figure 5). Its output is reversely transformed into the musculotopic space using the transformation weights **w** to drive the joint actuators. Functionally, precise output weights are critical, whereas the input weights may strongly vary without relevant loss in movement performance (Stratmann et al., 2016). Within the synergy space, a circuit as simple as a pool of excitatory reflex interneurons can control the movement (Stratmann et al., 2016). This control law is a promising hypothesis for neuronal motor control for three reasons: First, it requires information about the number of degrees of freedom prior to movement onset and thus minimum model knowledge. Second, it requires only information about muscular deflections and forces during an ongoing movement, as provided by proprioceptive fibers. Third, a linear ionotropic synergy circuit can implement this controller for unchanging environments. To adjust the control law to changing environments, multiplicative scaling of the neuronal gains **w** at the motor output stage is required, as will be explained in the following section. Since the number of degrees of freedom is the only required model knowledge, this control law is an ideal example to determine what minimal topographic precision serotonergic effects need at least in order to adjust synergies to changing contexts.

## 6. Multiplicative Gain Scaling Maintains Synergies in Changing Contexts

### 6.1. Gain Scaling Offers Unique Advantages to Neuronal Signal Processing

Mathematically, multiplicative gain scaling is a core principle for the extension of affine transformations. As will be shown, this principle can strongly enhance the presented robotic synergy controller. Thereby, it is possible to derive the spatial and temporal precision required by the serotonergic system to adjust synergic signal processing to changing contexts. Prior to that, it is important to consider how well multiplication can fulfill this task for realistic locomotor systems under arbitrary conditions. As will be shown, multiplication can in theory extend linear neuronal networks to fulfill this task arbitrarily well, because it allows them the implementation of arbitrary general transformations. Multiplication is furthermore a straightforward, functionally powerful operation for this task. These advantages of gain scaling are so fundamental that they apply to affine transformations in general, even beyond motor control. They motivate and help understand why precision scaling may have evolved during evolution.

Weierstrass and Stone (1948) have mathematically demonstrated that arbitrary continuous transformations *y*(*x*) can be approximated to any desired precision for a restricted interval of possible input values **x** by the sum of exponentiation powers in the input,

(6) $$\begin{array}{r} \text{y}\left(\text{x}\right)=+\left(-{\text{θ}}_{\text{y}}\right)+{\text{W}}_{\text{xy}}{\text{x}}^{1}+\ldots \;. \end{array}$$

Each summand comprises a power of the input with increasing exponent. Engineers often use this finding since this sum can be used to approximate arbitrary transformations which cannot be derived mathematically or are changing unpredictably with time. An affine transformation implemented by a linear neuronal network, as described by Equation (3), is a first-order approximation. That means it includes a constant, i.e., a term proportional to the zeroth power ***x***^0^ = **1** in the input, and a summand that is proportional to the first power ***x***^1^ in the input. Taylors theorem, one of the basic theorems in mathematical analysis, states that adding summands of higher exponent continuously improves the approximation. But given an approximation with summands up to a particular exponent, the benefit gained by adding further summands of higher exponent becomes increasingly negligible (Cohen and Tan, 2012). As affine transformations include terms up to the first power of the input, a linear neuronal network offers a general circuit scheme that captures a major portion of a general transformation. Linear neurons which are further able to multiply signals can be combined in several layers to calculate arbitrary powers of its inputs. In contrast to a purely linear neuronal network, such a network can implement Equation (6) and therefore perform each possible transformation on its inputs with arbitrary precision.

Deep learning shows that multiplication is only one out of many arithmetic operations which a neuronal network can implement in order to act as universal approximator of general transformations (Stone, 1948; Cybenko, 1989; Hornik, 1991; Chen et al., 2015). Hereby, the artificial networks typically implement a single function which seemingly resembles ionotropic signal processing, but may in fact represent the collective effect of ionotropic, metabotropic, and other mechanisms. While a multiplicative mechanism that parallels the ionotropic circuitry is not the only mechanism that allows implementing a universal approximator, it allows a particularly powerful, straightforward, and resource-efficient adjustment of an affine transformation to changing contexts. Adjusting an affine transformation,

$$\begin{array}{l} y_{j}=\underset{i}{\sum }w_{\text{ij}}x_{i}-\theta_{j}\;, \end{array}$$

according to the external signal *s* encoding the context implies that individual transformation weights *w*_ij_ must change with *s*. If the CNS multiplies the inputs *x*_*i*_ with the external signal, it effectively performs an affine transformation with transformation weights

(7) $$\begin{array}{r} w_{\text{ij}}\left(s\right)=w_{\text{ij},\text{c}}\cdot s\;, \end{array}$$

which increase with the constant of proportionality *w*_ij, c_. The resulting affine transformation

(8) $$\begin{array}{r} y_{\text{j}}=\underset{i}{\sum }w_{\text{ij},\text{c}}x_{\text{i}}s-\theta_{\text{j}} \end{array}$$

can be seen as a Taylor approximation which models the interaction between inputs *x*_i_ and context *s* up to second order. As explained in the previous paragraph, such a second-order approximation captures a large portion of an arbitrary interaction, which eases the functional need for further resource-consuming neuronal operations. In agreement with this functional benefit, experiments typically link changing coordinate transformations to gain modulation, as reviewed by Salinas and Sejnowski (2001). For example, motor output following stimulations of M1 is multiplicatively modulated by proprioceptive information (Graziano et al., 2004), which can at least partly be attributed to serotonergic gain scaling at the level of spinal motorneurons (Wei et al., 2014).

### 6.2. How Gain Scaling Can Enhance Synergy Control

In the specific context of robotic synergies, it is possible to derive the spatial and temporal precision that the spinal serotonergic system needs for precision scaling. Scaling the gains of the output transformation hereby leverages the above-described robotic control law, as it decouples the synergy circuitry from changes in the mechanical context of the movement (Lakatos et al., 2013). The synergy itself is therefore unaffected, for example, when one runs from a solid to a soft ground or changes body posture during cycling (cf. Figure 3). The common synergistic motor signal can be individually scaled by separate output gains *w*_j_ to calculate the respective motor signal for each functional group *j* of muscles acting on a single degree of freedom. A degree of freedoms is thereby typically formed by an individual joint (Lakatos et al., 2013, 2017). The index *i* of the synaptic weights *w*_ij_ is neglected, as the synergy circuit functionally outputs only a single ionotropic signal *x*.

Robotic control theory predicts how the gains *w*_j_ within the biological neuronal network must be adjusted to changing mechanical contexts in order to minimize metabolic demands (Lakatos et al., 2013). To explore a given mechanical context, the ionotropic synergy circuitry provides input to all involved muscles and excites a non-optimal movement. As the controller adapts to the mechanical context, it increasingly optimizes the movement. The control approach derived for this purpose (cf. Figure 5) resembles the function performed by the serotonergic feedback loop (cf. Figure 4) in all of its three main characteristics: First, the controller receives sensory input about the resulting joint deflections, resembling the proprioceptive information converging onto the raphe nuclei. Second, the controller uses this information to update its model of the body and its environment. For this purpose, it adjusts the transformation weights from the motor synergy to groups of actuators driving the involved joints. The updated transformation weights improve the movement and recursively lead to updated sensory signals. Also this characteristic resembles the function of the raphe nuclei, which scale ionotropic synergy signals as they arrive on motorpools. Third, the multiplicative transformation weights **w** converge toward the dominant principal component of the sensory signals **s** encoding the deflections of individual joints. Based on work by Lakatos et al. (2013), Stratmann et al. (2016) demonstrated that the alignment can be achieved by multiplying the output of the synergy circuitry by weights that increase with the sensory signals. In order to keep the weights within a physiological regime, decay of the weights over time is required as counteracting mechanism. These two effects can be summarized as

(9) $$\begin{array}{r} \frac{d}{dt}\text{w}=w_{\text{c}}\cdot \text{s}-\frac{1}{\tau }\text{w}\;. \end{array}$$

The constant factor *w*_c_ scales the overall force output. The time constant τ describes the gain decay and must be of the same order of magnitude as the typical cycle duration of biomechanical movement. This time scale guarantees constant gains throughout the movement cycle in a sustained context. Meanwhile, the dominant changes of transformation weights, and thus most of the functional impact on metabolic costs, occurs already during the first step cycles, i.e., for quickly-changing contexts (Stratmann et al., 2017). Stratmann et al. (2016) demonstrated analytically and in neuromechanical simulations that previous models of the kinematics of released serotonin are fully consistent with Equation (9). But it remained unclear if the serotonergic feedback loops shows the same temporal and topographic precision as the controller.

The resemblance between the serotonergic motor feedback loop and the controller is remarkable, as the controller has been derived based purely on considerations about the dynamics of biomimetic systems. After controller convergence, the synergy controller makes optimal use of the energy saving capacity offered by the elastic elements within the mechanical locomotor system. This result was consistently obtained under the influence of physical noise, mechanical damping, and non-linear dynamics (Stratmann et al., 2017). This means that the actuators require a minimum of metabolic energy to sustain the highly dynamic locomotion. Throughout the adaptation, the mechanical system shows stable movement. This stability results from the weight decay and from the friction within the mechanical system. The friction implements a further negative feedback loop as it increases with higher movement velocity, i.e., a stronger motor signal (Lakatos and Albu-Schäffer, 2014b). Videos illustrating the emerging movement have been published previously for elastic robotic systems mimicking the leg of a small mammal (Stratmann et al., 2017), human legs (Löffl et al., 2016), and a human arm (Lakatos et al., 2013). Under the assumption that the raphe nuclei show sufficient topographic and temporal precision, also the simulated raphe nuclei optimized the energy efficiency of motion induced in a leg which was either mechanically constraint, resembling cycling as illustrated in Figure 3, or free to move along a trail (Stratmann et al., 2016).

The robotics control approach explains the functional advantage of a raphe motor feedback loop that shows precision scaling rather than a diffuse neuromodulation of motorpools. Thereby, it predicts that the serotonergic feedback loop must show gain scaling which acts on a time scale of hundreds of milliseconds to few seconds and which is at least joint-specific. In particular, it must amplify motorpools driving joints that show a large deflection throughout the movement and thus send out large proprioceptive signals *s*_*i*_. With these characteristics, the raphe nuclei would ensure that simple ionotropic synergies can induce highly dynamic rhythmic movements with minimum metabolic demands under changing context.

## 7. Serotonin Provides Subcircuit-Specific Gain Scaling

The functional considerations offer a benchmark for the anatomical and functional precision that the serotonergic feedback loop requires to perform precision scaling.

**Figure 6:**
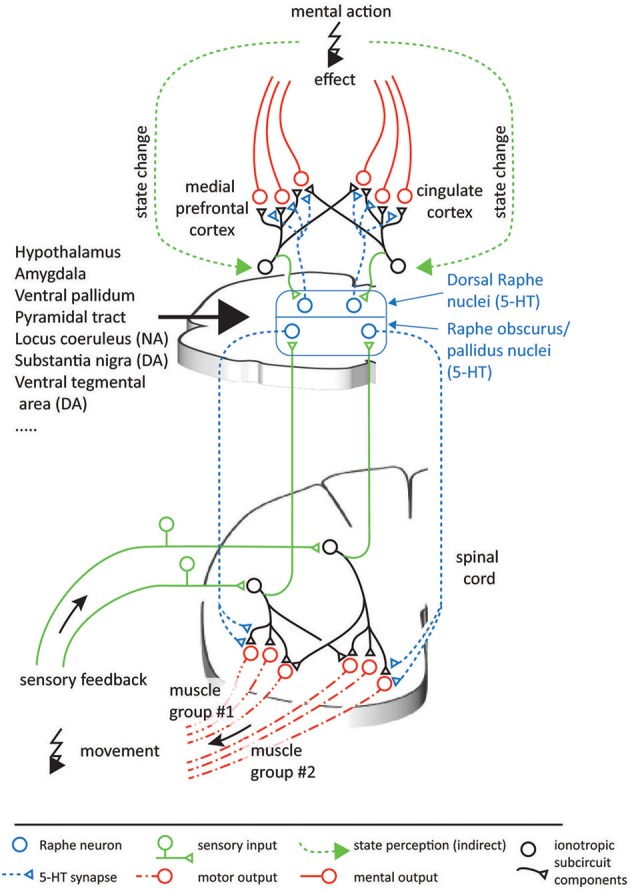
Possible analogous organization of serotonergic function in the spinal cord and the neocortex. In both CNS areas, populations of serotonergic projections originating in the raphe nuclei can be assumed to multiplicatively scale the output of individual ionotropic subcircuits. In turn, the projecting raphe neurons receive feedback on the outcome of their effects, thus forming feedback loops. The ionotropic signals in the spinal cord can be interpreted relatively easily, as they are directly linked to physical muscles and sensors. As in the case of the spinal circuitry, neocortical circuitry operations likely involve integration of information from groups of neurons, which are innervated by different combinations of serotonergic neurons. In contrast, the information encoded in the neocortex can be expected to be more abstract and less directly intelligible, and at present it is therefore less clear what coordinate spaces the neocortex combines. Nevertheless, as a first-level descriptive model of the functional mechanisms monoamines have in neocortical systems, the functional principle presented here can help understand the principles of the effects elicited by psychopharmacological drugs.

Neuroscientific studies considering the topographic precision suggest that along the serotonergic feedback pathway, each processing step allows for a spatially focused signal transduction. Sensory signals are relayed to the NRP and NRO within 20 ms (Springfield and Moolenaar, 1983). This short delay indicates a monosynaptic or a strong oligosynaptic input from the peripheral sensors to the NRO and NRP. A likely candidate is disynaptically mediated input via spinal interneurons that typically targets the cerebellum (Jörntell, 2017) but that may also mediate peripheral inputs to brainstem nuclei (Johansson and Silfvenius, 1977) as illustrated in Figure 6. The efferent serotonergic projections of the approximately 19,000 serotonergic neurons comprised within the human NRP and NRO (Hornung, 2003) target almost exclusively the ventral spinal cord (Martin et al., 1978; Loewy, 1981; Nieuwenhuys et al., 2008; Watson et al., 2012). These projections have been suggested to comprise both an anatomically diffuse component and a separate topographically specific component (Huisman et al., 2011). The diffuse projections as well as projections to ventral interneurons within a neuronal synergy affect the overall gain like the factor *w*_c_ in Equation (9) and may additionally increase the overall leg stiffness by co-contraction of antagonistic muscles. Both effects have been suggested to underlie increases of the movement speed (Heglund and Taylor, 1988; Ferris et al., 1998). They explain functional findings demonstrating that the 5-HT released by the action of one limb amplifies motor signals that target the muscles in other limbs as well (Wei et al., 2014). Previous anatomical studies allow a quantification of the spatially focused projection onto spinal motorneurons and interneurons associated with specific motorneuron pools. Tracers inserted into the spinal cord showed that the location of the labeled serotonergic cells vary markedly with the region of injection, contrasting the more homogeneous labeling of non-serotonergic cells within the raphe nuclei (Skagerberg and Bjorklund, 1985). Dual retrograde tracers injected into different regions of the ventral horn of rats double-labeled about 50% of cells within the NRP (Cavada et al., 1984). This degree of collateralization resembles that of corticospinal axons, for which more than 40% of 156 neurons could be activated antidromically from several segments of the spinal cord in monkeys (Shinoda et al., 1979). In the ventral spinal cord, serotoninergic projections predominantly terminate in synaptic contacts and the release of 5-HT shows effects of high spatial precision (Brumley et al., 2007; Cotel et al., 2013; Perrier et al., 2013). In agreement with a topographically precise spinal serotonergic system, depletion of 5-HT and blockage of 5-HT_2_ receptors in rats slackens locomotion due to adjustments in the motor signals which differentially affect muscles acting at different joints of the same limb or even the same joint (Myoga et al., 1995; Pflieger et al., 2002; Pearlstein et al., 2005). Evidence therefore suggests that the serotonergic system is able to induce effects which are at least joint-specific.

The time scale of metabotropic effects is slow in comparison to ionotropic signal transmission. Following sensory stimulation, the onset of the serotonergic multiplication effect was found to be delayed by tens of milliseconds after stimulus cessation in cats (Crone et al., 1988). It was shown to return back to baseline within a few seconds in turtles (Perrier and Delgado-Lezama, 2005), cats (Crone et al., 1988) and humans (Wei et al., 2014). This long time scale might impede fast neuronal calculations within the brain and may also have detrimental effects for motor control under rapidly changing conditions. For example, it may underlie the Kohnstam effect, where the arm involuntarily lifts following the abrupt end of a strong voluntary contraction. The Kohnstamm effect lasts for several seconds and originates in a persistent activation of the deltoid muscle, which is accompanied by higher motor evoked potentials. The underlying mechanisms are assumed to have a dominant spinal origin (Mathis et al., 1996; Ghosh et al., 2014). These properties are consistent with the idea that the excessive activity observed during the Kohnstamm effect is caused by serotonin that is released during a strong muscle contraction and increases the motoneuron gain of the deltoid specifically. Ongoing movements encountered in everyday life show less-abrupt and extreme switching between conditions. For such non-artificial movements, the time scale of serotonergic effects matches the time scale relevant to various motor behaviors.

To summarize, a joint-specific multiplicative effect which decreases on a time scale of seconds agrees well with the functional requirements determined for the stable and energy-efficient control of highly dynamic movement. The presented control-theoretical framework therefore links the previous experimental findings on monoamines into a new operational principle of temporally- and subcircuit-specific gain modulators. By this precision scaling, the serotoninergic projections to the ventral spinal cord can be assumed to strongly simplify motor control adaptation.

## 8. Monoamines Scale Signals Throughout the CNS

### 8.1. A Principle Common Across Serotonergic Systems

The previous section considered the parts of the serotonergic system that target the spinal cord. But the functional interpretation developed so far may, in principle, also apply to the serotonergic innervation of other parts of the central nervous system. It may even apply to those CNS areas which may achieve precision scaling using non-linear ionotropic mechanisms, possibly by combining many non-linear neurons into a network that approximates more general transformations mediated by network effects. These CNS areas may take advantage of the parallel, resource-efficient implementation of precision scaling originating from monoaminergic systems. Serotonergic innervation is present in practically all parts of the CNS, including the striatum, amygdala, thalamus, and hippocampus (Vertes and Linley, 2008; Daubert and Condron, 2010). But in this paper, a specific interpretational example will be developed for the frontal and cingulate areas of the neocortex, where many researchers locate at least part of the effects caused by psychopharmacological drugs interfering with the serotonergic system.

The proposed framework suggests a generic function for 5-HT as a subcircuit- and temporally specific non-linear gain modulator which scales individual weights of transformations between different processing stages by postsynaptic effects. An important component of this framework is formed by the feedback connections which evaluate the contextual conditions to update the drive on the serotonergic gain modulation (cf. Figure 4). Because of the subcircuit-specificity, there is differential gain scaling. This is useful if changes in conditions require the transformation of different aspects of the overall information to be multiplied with different factors to correctly interact with the external world. For the 5-HT innervation of the prefrontal cortex (PFC), most of these requirements seem to be confirmed. First, there are topographically precise projections with well-defined synapses from the nucleus raphe dorsalis (NRD) to the PFC (Bang et al., 2012; Belmer et al., 2017). Second, there is a topographically precise feedback inhibition from the PFC to the NRD (Jankowski and Sesack, 2004) and the NRD affects the neuronal gain in the layer V pyramidal cells of the PFC (Zhang and Arsenault, 2005). Considering these apparent functional homologies with the serotonergic innervation of the spinal circuitry, Figure 6 illustrates a possible scenario for the functional organization of the serotonergic innervation of the PFC / cingulate areas.

In the spinal cord, the functional principle proposed for 5-HT is the multiplicative scaling of individual transformation weights in order to adjust transformations between different coordinate spaces. Whereas for the spinal circuitry one can speak in relatively concrete terms of what is being represented and on what bases the coordinates exist, the coordinates relevant to integrative neocortical systems are likely to have much more abstract bases and are anyway not well-known at the moment. However, there are studies of correlations between certain abstract measures and the activity of the neurons, which can serve as approximations of what kinds of representations are involved. Primate PFC neurons can encode at least in part the monitored actions (Yoshida et al., 2011) and the errors of action of other monkeys (Yoshida et al., 2012). In the anterior cingulate cortex, neurons strongly respond to rewards delivered to other monkeys, while orbitofrontal neurons are more biased toward rewards delivered to the recorded monkey (Chang et al., 2013). An effect of lesions in the orbitofrontal cortex is abnormal social and emotional judgements of facial expressions (Willis et al., 2010; Watson and Platt, 2012) possibly associated with autism or sociopathy (Chang et al., 2013). In rodents, an optogenetic stimulation of PFC neurons that project to the NRD creates abnormal avoidance behavior (Warden et al., 2012; Challis et al., 2014).

Consider the possibility that the neocortex, as we envisaged for the spinal circuitry organization around synergy control, consists of multiple subcircuits, or groups of neurons. Each subcircuit carries representations of specific parameters which are directly or indirectly relevant to dealing with situations arising mentally or in the social world. Because the subcircuits interact, the optimal weighting of each subcircuit will depend on context, similar to the relative muscle forces needed for locomotion across different terrains. In this case, the serotonergic system may scale the relative contributions of different subcircuits so that their contributions to the output become proportional to the required contributions which are imposed by the situation (cf. Figure 6).

Attempts toward more holistic models of the functional role of 5-HT have emerged from studies on lower animals, such as the lobster (Kravitz, 2000). In the social life of the lobster, 5-HT levels are assumed to gradually build up during encounters with other lobsters. Encounters typically end up in a gradually escalating demonstration of power in which the lobster with the most imposing body language, or, more rarely, physically proven superiority, will maintain high 5-HT levels and an imposing body posture. Conversely, the individual losing the social tete-a-tete will rapidly develop a subordinate body posture which is assumed to be associated with a dramatic decline in 5-HT levels. The subordinate will subsequently avoid engagement in social fighting for a long time. This acquired unwillingness to engage in fighting can be discharged, however, by an experimental manipulation of the 5-HT levels (Kravitz, 2000). In this model system with a low degree of behavioral diversification, 5-HT will hence affect social interactions and the level of 5-HT will also be a consequence of the behavioral outcome on the social stage. In mammals, possessing a more highly developed neocortex and hence a more diversified and richer understanding of the external world, one would expect a more complex set of feedback regulations of the serotonergic system. Still, the serotonergic system may abide the same principle, i.e., the serotonin level is a consequence of the actions we take and the effects we perceive them to produce. A high or a low level of 5-HT is not necessarily good or bad, but the level should rather be appropriate for how we perceive our position with respect to the external world.

Applying this type of functional model of 5-HT actions also to mental brain functions can offer a novel interpretational framework for the action of psychopharmacological drugs linked to malfunction of the monoaminergic system. Associated disorders include depression, melancholia, social anxiety disorder, obsessive compulsive disorders, panic disorders, posttraumatic stress syndrome, and generalized anxiety disorder. Drugs which are used against these disorders and interact with serotonin and monoamine synaptic transmission are sometimes viewed as pharmaceutical pushbuttons for specific emotional qualities, even though there seems to be no good support for assuming direct causality (Ruhe et al., 2007). However, as portrayed above, the cortical systems that can be expected to be ultimately responsible for the perception of our mood appear to provide feedback projections permitting them to regulate their own 5-HT release (Peyron et al., 1998). As in every negative feedback system, a set point of activity that the system strives toward will tend to arise. Temporary variations around that set point can be triggered by novel estimates of the prevailing conditions based on inputs from the ionotropic circuitry. Hence, according to this view, psychiatric disorders that are susceptible to treatment with drugs interfering with the 5-HT system may arise when the multiplicative coordination of activity for different subcircuits have fallen outside their normally functioning set points. If the scaling of the relative contributions of different subcircuits carrying mental models is out of order, the responses to a changing environment would become inadequate, which may start a vicious circle in which the system digresses further away from its functional set points. In principle, this could occur as a consequence of behavior and would thus be acquired, although internal predisposition factors could exist as well. Interference with 5-HT transmission by the clinical administration of seletive serotonin reuptake inhibitors (SSRIs) could theoretically push the set points of 5-HT in the different subcircuits to new ranges. In some patients, these emerging ranges turn out to be functionally operative. In many cases, however, the doses need to be individually adjusted over a long time. And for some patients SSRI treatment does not work irrespective of dose (Rush et al., 2006; Trivedi et al., 2006). Another feature of SSRI treatment that seems to indicate the existence of internal set points for 5-HT activity is that the therapeutic effect of SSRI is often delayed by some two weeks. One part of this delay has been hypothesized to be due to the autoreceptors on the synaptic terminal that releases serotonin (Richardson-Jones et al., 2010). The autoreceptors exert a negative feedback on the amount of serotonin released by the terminal and thus forms another natural negative feedback system. However, a negative feedback acting across such a short diffusion range and with effects isolated to the own terminal would seem unlikely to normally take two weeks to find a new set point. But the long-range feedback connections back to the raphe nuclei, involving subcircuits of ionotropic neurons, where each neuron may be expected to have a differentially time-varying activity across different conditions, could well result in feedback systems with very long time constants. Hence, they seem to be a more logical explanatory model for such extremely delayed effects.

### 8.2. A Principle Common Across Monoaminergic Systems

Other monoaminergic systems than the raphe nuclei also function according to principles that strongly resemble the precision scaling function that is proposed here for serotonin in the spinal cord.

A beautiful and perhaps unexpected example comes from the apparent function of the dopaminergic innervation of the retina (Bargmann, 2012). Retinal processing is dominated by cone photoreceptors in bright light and by rod photoreceptors in low light. Both sensor types converge on cone bipolar cells, which receive direct input from cones as well as indirect input from rods relayed through intermediate rod bipolar cells and AII amacrine cells. When the light level is high, the responsible dopaminergic neurons are activated (Brainard and Morgan, 1987) and the gap junctions between AII amacrine cells and cone bipolar cells are uncoupled. This uncoupling is triggered by the action of dopamine at gates exclusively on the amacrine side, implying that it does not interfere with the processing of inputs from the cone photoreceptors (Xia and Mills, 2004). Uncoupling can be considered to be a multiplicative effect, in which the aim is to find the relative scaling that gives the best overall information for the current light level (Mills and Massey, 1995; Xia and Mills, 2004; Bargmann, 2012). This function is akin to the proposed effect of 5-HT in the ventral spinal cord, which scales the relative motor signals actuating individual groups of muscles according to sensory signals in order to optimize the overall force output.

In general, most monoaminergic systems share the principal features that underlie the model of serotonergic precision scaling presented in this paper. In particular, they are under the tight control of the hypothalamus (Veazey et al., 1982; Villalobos and Ferssiwi, 1987). In some cases, they are even part of the hypothalamic nuclear complex (Ugrumov, 1997), as for example the tuberomammillary nucleus of the hypothalamus in case of histaminergic neurons (Haas et al., 2008). The monoaminergic systems send dense projections to each other, suggesting that their respective activities are under mutual control (Ericson et al., 1989; Nakamura, 2013). They all have widespread terminations in most major structures of the CNS (Samuels and Szabadi, 2008; Vertes and Linley, 2008; Daubert and Condron, 2010; Nestler et al., 2015; Yu et al., 2015). They receive feedback connections from the structures they target and they have autoreceptors for the local feedback of their synaptic release (Douglas et al., 2001; Garcia et al., 2004; Richardson-Jones et al., 2010; Ford, 2014). The bulk of their projections go to the ionotropic circuitry where they act primarily by changing conductances which modulate gains (Foehring et al., 1989; Dong and White, 2003; Surmeier et al., 2007; Yu et al., 2015) in the targeted neurons. Among others, targets include the neocortex, thalamus, striatum, cerebellum, hippocampus, and amygdala. In many cases, there is support for a subcircuit-specific regulation (Blandina et al., 2012).

### 8.3. A Principle Preserved Across Phylogeny

The presented evidence suggests that precision scaling fundamentally extends the functions of the ionotropic circuitry. Therefore, it comes as no surprise that the monoaminergic systems have emerged very early in phylogeny (Parent, 1984) and that their effects have often been strikingly preserved in the course of natural selection. The serotonergic motor feedback loop, which we describe in detail for mammals, can for example be traced back to invertebrates. Also in these animals, serotonergic neurons strongly innervate motor circuits and receive corresponding feedback (Gillette, 2006). Once serotonin is released, motoneurons show equal reactions across species boundaries and increase their gain in Aplysia (Mackey et al., 1989) as well as in cats (Crone et al., 1988) and humans (Wei et al., 2014). In the lobster, it is known that serotonin can act with topographic precision and specifically increase the firing of flexor muscles. This increased flexor excitation induces the imposing body posture which was described above for dominant lobsters (Kravitz, 2000). Similar to the serotonergic motor feedback loop described here, the amacrine dopaminergic system in the retina has been found also in cartilaginous fishes and amphibians (Yamamoto and Vernier, 2011). A difference from the spinal circuitry is that the topographic precision of the population-integrated dopaminergic projection to the retina is not achieved by the distribution of presynaptic terminals and their amplification of ionotropic currents, but by acting on gap junctions. This reflects that gap junctions play a major role in retinal signal processing (Bloomfield and Volgyi, 2009), whereas the influence of electrical coupling within the spinal cord strongly decreases with developmental age (Li and Rekling, 2017). Thus, it is likely that precision scaling has independently emerged in different CNS regions based on the biochemical mechanisms that dominate the respective signal processing.

## 9. Experimental Predictions and Conclusions

In order to test if the CNS takes advantage of monoaminergic precision scaling, it is most convenient to investigate the serotonergic motor feedback loop implemented by the raphe nuclei. For this circuit, the control of biomimetic robots clearly predicts the hypothesis that must be evaluated: The excitability of a motorpool actuating a specific joint must increase primarily after subjects have moved the respective joint rather than other joints of the same limb. As serotonergic effects on motoneurons remain for several 100 ms, the increased excitability must be observable also after cessation of the movement and the motor signals that drive it. Given this predicted topographic precision, the raphe nuclei can adapt motor control to changing conditions and ensure highly-dynamic locomotion under minimum metabolic demands.

While this paper elaborates subcircuit-specific neuromodulation mainly for spinal circuitry, precision scaling presents a big picture which frames the ubiquitous monoaminergic neuromodulation across the CNS. Accordingly, monoaminergic systems represent a computational network within the network formed by the ionotropic circuitry. While subcircuits can collectively encode predictive models of the world, monoamines adapt these models to contextual changes by scaling the ionotropic output signals. This concept offers an attractive explanation of how metabotropic signal processing complements the ionotropic functional and anatomical connectome: By scaling individual ionotropic signals, monoamines can provide functionality that is powerful, resource-efficient and, at least in the spinal circuitry, unique. Furthermore, the slow time scale of metabotropic effect coincides with the time scale of many motor behaviors, rendering monoamines ideal candidates to bridge the fast ionotropic signals and slowly changing behavioral context. In turn, the long time scale of metabotropic mechanisms can impose testable detrimental limits on the speed of behavioral adaptation, as exemplarily observed in the persistent impairment of precision movements resulting from serotonergic effects after high muscular force production (Wei et al., 2014). Given these facts, it is not surprising that malfunction of monoaminergic systems is strongly implicated in motor and cognitive disorders. Conceptualizing monoaminergic systems as subcircuit-specific modulators of ionotropic circuitry thus helps scale our view on why diffuse psychopharmacological drugs often show unpredictable treatment outcomes in such disorders.

## Author Contributions

AA-S, HJ, and PS developed the structure of the paper. PS authored sections 1–7 and 9. HJ authored section 8. AA-S, HJ, and PS critically revised the paper.

### Conflict of Interest Statement

The authors declare that the research was conducted in the absence of any commercial or financial relationships that could be construed as a potential conflict of interest.
